# Estimated energy and nutrient intake for infants following baby‐led and traditional weaning approaches

**DOI:** 10.1111/jhn.12981

**Published:** 2022-01-27

**Authors:** Hannah Rowan, Michelle Lee, Amy Brown

**Affiliations:** ^1^ School of Health and Social Care, Faculty of Medicine, Health and Life Sciences Swansea University Swansea South Wales UK; ^2^ School of Psychology, Faculty of Medicine, Health and Life Sciences Swansea University Swansea South Wales UK

**Keywords:** baby‐led weaning, complementary feeding, energy intake, infant feeding, nutrient intake, weighed diet diary

## Abstract

**Background:**

Baby‐led weaning (BLW), where infants self‐feed without the use of spoon‐feeding by a caregiver, continues to be a popular approach for starting solids. However, concerns remain amongst health professionals that infants using this method may not consume sufficient energy or nutrients from solid foods. Little research has examined how different weaning approaches shape dietary intake. The present study aimed to use a 3‐day weighed diet diary to measure estimated energy and nutrient intake in infants aged 6–12 months.

**Methods:**

Diet diaries were completed by 71 parents and analysed to compare estimated infant intake from milk and solid foods for those either following a BLW or traditional spoon‐feeding approach (TW). Intake was analysed for each weaning group in two age groups: 26–39 and 40–52 weeks, to account for different eating patterns at the start and end of the weaning process.

**Results:**

For the younger infants, significant differences in estimated energy intake were found, with TW infants consuming 285 kcal from solid foods compared with 120 kcal for BLW infants. Conversely, BLW infants consumed more calories and nutrients from breast or formula milk, consistent with a slower transition to solid foods. No differences were found in estimated intake amongst older infants, suggesting that BLW infants had ‘caught up’ with their spoon‐fed peers.

**Conclusions:**

Overall, few infants regardless of weaning group met recommended intake guidelines for energy (either over or under consuming) with many deficient in iron and zinc intake. The findings are important for those supporting parents through the transition to solid foods.

## Key points

- At 6–9 months, infants following a baby‐led weaning (BLW) consumed less energy from solid foods compared to traditionally weaned (TW) infants with over three‐quarters of TW infants consuming more than World Health Organisation (WHO) guidelines.

- Infants following a BLW approach had a more gradual transition to solid foods than those following a TW approach more closely supporting WHO guidelines. However, some parents may need further support with respect to offering more solid food exposures. No differences were found in energy, macro‐ or micronutrient intake for infants aged 10–12 months for infants following BLW or TW approaches, suggesting a convergence of intake towards later infancy.

- Many infants, regardless of weaning approach, did not meet the recommended guidelines for iron or zinc intake, suggesting that there is a need to focus on promoting the intake of these micronutrients.

## Graphical abstract

Infants following baby‐led weaning have a slower transition to solid foods, which may be protective against overeating

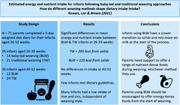

## INTRODUCTION

Baby‐led weaning (BLW), where infants self‐feed solid foods rather than being traditionally weaned (TW) using spoon‐fed purees, remains popular in the UK. Parents are often drawn to BLW because they consider that it will promote healthier eating behaviour and weight,^1^ but some health professionals are concerned that it may promote undernutrition.^2^, ^3^ Although research exploring the impact of BLW is building particularly around eating behaviour and weight,^4^ evidence is often based on parental report, with a clear gap in our understanding of its impact upon infant dietary intake, particularly in the UK.

In New Zealand, the Baby‐Led Introduction to SolidS (BLISS) randomised controlled trial of a ‘baby‐led’ vs. TW approach upon child weight and intake, as assessed using a weighed 3‐day food diary, utilised a modified form of BLW and offered a wider variety of high energy and iron rich foods.^5^ Baby‐led infants consumed less saturated fat at 12 months, but no difference was found by 24 months.^6^ Meanwhile, for micronutrient intake, there was no significant difference in zinc or intake at 7 and 12 months.^7^ Overall, although energy intake was similar, baby‐led infants consumed more sodium, but less iron, zinc, calcium, vitamin C, vitamin B_12_ and fibre, than TW infants.^8^ Another trial in Turkey compared iron intakes and serum levels from 280 infants following BLW or spoon‐feeding. No differences were found between weaning groups at 12 months for serum iron markers or iron consumption; however, iron intake in both groups was lower than the Turkish recommended daily allowance for infants aged 12 months.^9^

In the UK, three studies have explored nutrient intake between BLW and spoon‐fed infants using a 24‐h dietary recall. One study found no significant difference in energy, carbohydrates, protein saturated fat or zinc intake,^10^ but found the spoon‐fed group consumed more free sugars at 6–8 months of age, whereas the BLW group consumed more fat and sodium. Neither group met the reference nutrient intake (RNI) for iron but the BLW consumed significantly less than the spoon‐fed group. Another study found several significant differences between weaning groups,^11^ with strict BLW infants being more likely to consume vegetables and protein foods than TW infants at 6–8 months, less likely to have dairy at 9–10 months, and less likely to have savoury snacks, dairy and composite meals at 11–12 months. There was no difference between weaning groups in consumption of iron‐containing foods.

In the third recent study, infant intake was estimated using a multiple‐pass researcher led 24‐h recall.^12^ In this type of study, participants are asked to recall the diet for the day before details of individual foods/drinks, portion sizes and recipes are collected. This method collects a greater detail and depth of intake, allowing energy and micronutrients to be estimated more accurately. Overall, 96 mother–infant dyads completed the 24‐h recall (60 TW and 36 BLW). Although no difference in energy intake was found between the weaning groups, BLW infants consumed more energy from milk and TW infants more energy from solid foods at 6–8 months. At 6–8 months, TW infants consumed higher levels of iron, zinc, iodine, vitamin B_12_ and vitamin D than BLW infants, although 44% of TW and 63% of BLW consumed below the lower reference nutrient intake for iron. However, most differences disappeared by 9–12 months, when most infants had transitioned to self‐feeding and eating a family diet. There were few differences in food exposure between the groups, although TW infants consumed higher levels of commercial products.

However, there are limitations to 24‐h recall, such as participant error, failure to remember quantities of food correctly or desire to alter reported food intake. Given the sparsity of research in this area, coupled with concerns regarding nutrient intake expressed by health professionals,^2^, ^3^ the present study aimed to conduct a detailed examination of estimated infant nutrient intake, comparing those following a BLW or TW introduction to solid foods, using a 3‐day weighed diet diary. Specifically, the aim was to compare whether overall estimated energy, macronutrients and micronutrients differed between the two weaning approaches at the start (26–39 weeks) and end (40–52 weeks) of the weaning process.

## METHODS

### Participants

Parents with an infant aged 6–12 months were eligible to complete the study. All respondents were female, aged ≥18 years, living in the UK, and had started the weaning process. Exclusion criteria included infant prematurity (gestation < 37 weeks), low birth weight (<2.5 kg) and multiple food allergies, failure to thrive or other complex health issues that might affect diet.

Approval for this study was granted by Swansea University College of Human and Health Research Ethics Committee. All mothers gave informed consent prior to inclusion in the study.

### Measures

Alongside a 3‐day weighed food diary for their infant, respondents completed a questionnaire, including demographic background (age, sex, education and employment status), infant characteristics (sex, age in weeks and parent‐reported weight) and the method of introducing solid foods.

They were also asked how they identified with the following statement in terms of how closely they were following a baby‐led method of introducing solid foods: strictly, loosely, not at all:

*BLW is the process of placing foods in front of your baby and letting them feed themselves – picking the food up themselves and putting it in their mouths unassisted, rather than being spoon‐fed by a parent. This could involve them using a spoon themselves. BLW tends to involve offering the baby family foods rather than offering pureed foods*.

This self‐identification was then verified by asking two follow up questions on how frequently they used spoon‐feeding with their infant or used purees. Each scale had a seven‐point response option from 100% spoon feeding/puree use to 100% self‐feeding/whole foods.

Respondents were asked to complete a weighed 3‐day food diary. Weighed food records involve recording the weight of each item before eating, then weighing leftovers to provide an accurate picture of what is ingested rather than offered. From this record, an assessment of the caloric and nutrient content of the diet is made. Weighed food records are considered an accurate measure of estimated energy intake and more reliable than food frequency questionnaires and 24‐h recall.^13^ They have been validated as being comparable to physiological measures of energy intake, such as the doubly labelled water method,^14^ and have been used in a number of studies examining estimated nutrient intake in infants^15^, ^16^ including the BLISS study.^17^ However, they do have limitations, including a degree of under‐reporting, which we consider in the discussion.

Parents were asked to weigh and note all of the foods they gave their baby over three selected days, which did not have to be consecutive. Parents were asked not to complete diaries when their child was at day care as a result of the practical limitations for childcare workers completing the diary, introduction of another participant into the study and the risk of inaccuracies between different individuals completing the diaries.

To complete the weighed food diary, parents were provided with portable but accurate scales (Salter Arc 1066l accurate to 1 g), which have been used in similar research.^11^ To record each entry, parents were given detailed instructions about how to weigh each food offered and how to record brand, type, preparation and consistency: pureed, mashed, chopped or whole (Figure 1). Parents were asked to weigh leftovers, whether dropped on the floor or in a bowl or bib, although the complexity of this and its impact on intake accuracy is recognised. Breastfeeding mothers were asked to estimate the number and duration of feeds, whereas those formula feeding were asked to note the amounts offered and remaining in the bottle after each feed.

### Procedure

Adverts for the study containing brief information, inclusion criteria and researcher details were shared online in baby and feeding groups on social media and in local baby groups. Potential participants contacted the lead researcher and were given full study information. Those who wished to take part and met inclusion criteria were sent a study pack containing scales, demographic questionnaire, consent form and diet diary. A debrief at the end of the diet diary encouraged participants to seek advice from a healthcare provider if the survey had raised any concerns alongside a reminder of researcher contact details.

### Data analysis

The initial data analysis plan included analysing three types of weaning approach (strict BLW, loose BLW and TW), which had been used in previous research^11^ for two infant age groups (26–39 and 40–52 weeks). However, as the diaries and forms clarifying feeding style were returned, it was clear that two main weaning groups were emerging: a stricter BLW approach and an approach based on a mixture of self and spoon feeding. Given that the UK Department of Health guidelines now encourage finger foods alongside purees, the decision was made to switch to two main weaning groups for analysis. The final sample size was similar to those used in previous New Zealand studies.^5^, ^8^

#### Measuring intake

Diet diaries were analysed using Nutritics dietary analysis software (Nutritics Professional Plus, version 5.099; Nutritics), which uses multiple nutrition databases including the UK Composition of Foods Integrated Dataset (COFIDS).^18^ Generic food items can be entered individually (e.g., 50 g of banana), although the database contains many branded items with information supplied by the manufacturer. If using homemade meals, parents were asked to supply a recipe, which was manually entered, using standard ingredients listed in the database, such as pasta, tomato sauce and courgette. The weight change factor in Nutritics was applied if appropriate. This function changes the data for foods that have been cooked to take into account any weight changes and nutrient losses during preparation. For example, vitamin and mineral content in particular might reduce during the cooking process.

If not supplied, the researcher used the database's standard meal function, for example, ‘homemade tomato and vegetable pasta sauce’ or, if a branded product not listed in the database was reported, the researcher manually created a new database entry using the manufacturer's standard nutrition labelling, including calories, carbohydrates, protein, fats, sugars, fibre, sodium and other nutrients, if stated. Branded infant food data already included in Nutritics were used even if incomplete, rather than being substituted for alternative foods by the researcher to minimise errors in nutrients already assessed by the manufacturer.

The majority of diet diaries contained specific weights for foods offered, but, where parents had used less accurate portion sizes (e.g., a tablespoon), the weights reported in Nutritics for these items were used. Clearly, the nutritional data from these meals is not as accurate as it might have been if the participant had supplied a recipe, but, given the small quantities of foods often eaten by infants and the similarity of many common, family style recipes, this was an acceptable substitute.

Finally, to measure the total amount eaten, leftovers were subtracted from the amount offered. For mixed meals where it was hard to remeasure individual ingredients left by the infant, remainders were recorded in proportion with the amounts offered.

#### Measuring breastmilk intake

Measuring breastmilk intake is complex. Potential options include weighing before and after feeds or measuring salivary/urine isotopes, which are accurate methods, but impractical for such a study.

A more practical method in similar research has used infant age to estimate intake based on studies that have calculated intake figures from total breast milk consumption measured by test weighing or stable isotopes.^15^, ^19^ This approach was used by the BLISS study to estimate breastmilk intake in infants age 7 and 12 months.^17^ Using this method, they included quantities of 708 g of milk per day for 26–39 weeks and 547 g for 40–52 weeks.^20^ Although, initially, we hoped to calculate breastmilk intake by asking mothers to record frequency and duration of feeds, as a result of limitations of this approach and incomplete maternal data, we chose to adopt the same methodology used in the BLISS study.

#### Analysing overall intake

A Nutritics report was generated for the average estimated intake over three days for overall energy intake, macronutrients (e.g., carbohydrate, fat) and micronutrients (e.g., iron, zinc). Estimated intakes were compared for the two weaning groups for infants aged 26–39 and 40–52 weeks to represent the earlier and later stages of weaning. Intakes were calculated for solid foods alone and then solid foods plus milk.

Estimated intakes were also examined where possible in relation to dietary reference values from the World Health Organisation (WHO) or UK Scientific Advisory Committee on Nutrition (SACN) infant intake recommendations. However, for infants under aged <2 years, there are no official recommendations for carbohydrates, sugar or fibre (or fats below 5 years of age) because of a lack of data on optimal intakes. The RNI is the average daily intake of a nutrient sufficient to meet the needs of 97.5% of a healthy population. Energy intake was considered in relation to the estimated average requirement (EAR). The EAR for energy or a nutrient is the mean intake that a group of people will need, with half of a defined population usually needing more than the EAR, and half less.

Statistical analysis was performed using SPSS, version 25 (IBM Corp.). Differences in demographic background by weaning group were analysed using *t* tests or the ch‐squared test. Only a difference in timing of solid foods was found and therefore used as a covariate throughout analyses. We tested the distribution of estimated nutrient intakes for normality using Kolmogorov–Smirnov tests and found all measures to be skewed. Nutrient data was therefore transformed and the natural logarithms computed used to correct for the skewed distribution. Multivariate analysis of covariance (MANCOVA) was then used to compare estimated energy, macronutrient intake and micronutrient intake between the two weaning groups, with separate analyses for the two age groups. Analyses were conducted considering estimated intake from solid food alone followed by intake combining both solids and milk foods. We used the non‐transformed data to present median intake scores for this to be logically comparative with other studies.

## RESULTS

Seventy one mothers completed the study, with a mean (SD) age of 32.8 (5.0) years, ranging from 22 to 43 years of age. Infants in the study ranged from 27 to 52 weeks of age, with a mean (SD) age of 40 (7.9) weeks; 35 were female and 36 male. Full demographic information for mothers taking part is shown in Table 1.

**Table 1:** Maternal demographic information: whole sample

		Whole sample		Baby‐led weaning		Traditional	
Indicator	Subgroup	*n*	%	*n*	%	*n*	%
Maternal age (years)	22–24	3	4.2	2	7.7	1	2.2
	25–29	16	22.5	3	11.5	13	28.9
	30–34	24	33.8	10	38.5	14	31.1
	35–39	22	31.0	8	30.8	14	31.1
	40–43	6	8.5	3	11.5	3	6.7
Education level	GCSE	2	2.8	1	3.8	1	2.2
	A Level	11	15.5	5	19.2	6	13.3
	Degree or equivalent	23	32.4	7	26.9	16	35.6
	Postgraduate or equivalent	35	49.3	13	50.0	22	48.9
Marital status	Married	49	69	17	65.4	32	71.1
	Widowed	–	31	9	34.6	13	28.9
	Divorced	–					
	Separated	–					
	Living with partner	22					
	Single	–					
Employment status	Full time	4	5.6	–	–	4	8.9
	Part time	14	19.8	4	15.4	10	22.2
	Maternity leave (will return)	40	56.3	15	57.7	25	55.6
	Maternity leave (won't return)	7	9.8	2	7.7	5	11.1
	Not working	6	8.5	5	19.2	1	2.2
Infant feeding style	Breastfeeding	49	69.0	23	88.4	26	57.8
	Formula feeding	12	16.9	2	7.8	10	22.2
	Mixed feeding	8	11.3	1	3.8	7	15.6
	Expressed breast milk	1	1.4	0	0.0	1	2.2
	Cow's milk	1	1.4	0	0.0	1	2.2

Twenty six infants were introduced to solids in a strict BLW manner, whereas 45 followed a TW approach. Thirty five infants were in the 26–39 week group (14 BLW/21 TW) and 36 in the 40–52 week group (12 BLW/24 TW). No significant differences in maternal age, education, marital status or employment were found between weaning groups. For infants, no significant differences in sex or age in weeks were found between weaning groups. However, infants in the BLW group were introduced to solid foods at a mean (SD) age of 25.4 (1.5) weeks compared to 24.3 (2.8) weeks in the TW group (*t*_66.080_ = 2.314, *p* = 0.024).

When considering parent‐reported infant weight, there was no significant difference in between weaning groups at either age group. In the 26–39 week age group, the strict BLW group had a mean parent‐reported weight of 8.6 kg, whereas the TW group weighed an average of 8.4 kg. In the older group (40–52 weeks), the mean weight of the strict BLW group was 9.6 kg, whereas the TW group mean was 9.8 kg. None of the infants was underweight according to the WHO centile charts for age/weight.

As shown in Table 1, with regard to milk feeding in the BLW group, 23 mothers were breast feeding, two used formula and one used mixed feeding. In the TW group, 26 were breastfeeding, 10 used formula and seven used mixed methods. After excluding two infants fed using expressed breast milk and cow's milk in the older age group, when comparing milk feeding methods using a chi‐squared analysis, there was a significant association between milk feeding style and weaning group (χ^2^ = 6.205, *df* = 2,69, *p* = 0.045), with BLW infants more likely to be exclusively breastfed.

### Age group 1: 26–39 weeks old

Differences in overall estimated energy intake, macronutrients and micronutrients were analysed using MANCOVA controlling for timing of solid foods. As shown in Table 2, for solid foods alone, infants in the TW group had a median estimated intake 2.2 times that of the BLW group, which was significantly different (*p* < 0.001). This gap reduced but was still significant when milk was taken into account. In both age groups, BLW were more likely to eat under the WHO EAR and TW infants over the WHO EAR.

**Table 2:** Comparison of estimated energy intake between weaning groups from solid foods and milk at 26–39 weeks using multivariate analysis of covariance

		WHO EAR	Median intake (25th, 75th percentile)	% < WHO EAR	% > WHO EAR	Comparison of energy intake between groups
Solid food	BLW (*n* = 14)	820 kJ	506.0 (280.0, 678.0) kJ	13 (92.9%)	1 (7.1%)	*F*_1,32_ = 11.102, *p* < 0.001
		196 kcal				
			121.0 (69.0, 162.0) kcal			
	TW (*n* = 21)		1117.0 (839.0, 1498.0) kJ	5 (23.8%)	16 (76.2%)	
			267.0 (200.5, 358.0) kcal			
Solid food + milk	BLW (*n* = 14)	2855 kJ	2607.0 (2268.0, 2751.0) kJ	12 (85.7%)	2 (14.3%)	*F*_1,32_ = 6.448, *p* = 0.04
			623.0 (542.0, 657.5) kcal			
		682 kcal				
	TW (*n* = 21)		3105.0 (2701.0, 3333.0) kJ	6 (28.6%)	15 (71.4%)	
			742.0 (645.5, 796.5) kcal			

Abbreviations: BLW, baby‐led weaning; EAR, estimated average requirement; TW, traditional weaning; WHO, World Health Organization.

Table 3 shows the mean estimated macro‐ and micronutrient intake for infants in the two weaning groups. For macronutrient intake for solid foods alone, a number of significant differences were found. TW infants consumed significantly more carbohydrate, fibre and protein than the BLW infants, although most of these differences disappeared once milk was also taken into account.

**Table 3:** Comparison of estimated nutrient intake between groups from solid foods alone and solid foods and milk at 26–39 weeks using multivariate analysis of covariance and transformed data

		Solids foods only			Solids and milk		
		BLW (*n* = 14)	TW (*n* = 21)		BLW (*n* = 14)	TW (*n* = 21)	
	RNI	Median (25th, 75th percentile)		*p*	Median (25th, 75th percentile)		*p*
Carbohydrate (g)	No RNI < 2 years	12.6 (7.9, 18.0)	36.3 (28.2, 48.0)	*p* < 0.001	63.5 (54.7, 70.0)	82.0 (77.0, 94.5)	*p* < 0.001
Protein (g)	12.7–13.7^a^^,^ b	4.6 (2.5, 5.9)	9.7 (7.7, 15.6)	*p* < 0.001	13.9 (11.7, 15.9)	18.9 (17.1, 23.8)	*p* < 0.001
Fat (g)	No RNI < 5 years	4.9 (2.6, 6.1)	8.5 (4.8, 11.7)	*p* < 0.005	34.4 (31.0, 35.1)	34.41 (31.9, 37.3)	*p* = 0.774
Saturated fat (g)	No RNI < 5 years	1.6 (0.7, 2.5)	2.9 (1.6, 4.2)	*p* = 0.011	15.5 (14.1, 16.2)	15.1 (14.0, 16.8)	*p* = 0.662
Sugar (g)	No RNI < 2 years	3.4 (1.8, 7.5)	12.8 (7.8, 17.4)	*p* < 0.001	53.0 (50.0, 56.5)	59.0 (53.5, 64.0)	*p* = 0.249
Free sugars	No RNI < 2 years	1.0 (0.4, 2.4)	1.6 (0.5, 3.6)	*p* = 0.03	0.4 (0.1, 1.0)	1.6 (0.5, 3.6)	*p* = 0.03
Fibre (g)	No RNI < 2 years	1.8 (1.1, 2.2)	4.0 (3.4, 6.1)	*p* < 0.001	2.1 (1.2, 2.5)	5.5 (3.6, 6.5)	*p* < .001
Iron (mg)	4.3–7.8^a^^,^ b	0.6 (0.4, 7)	2.0 (0.8, 2.7)	*p* = 0.005	1.1 (0.91, 1.5)	3.0 (1.7, 5.8)	*p* = 0.003
Zinc (mg)	5^b^	0.5 (0.3, 7)	0.81 (0.4, 1.5)	*p* = 0.058	2.6 (2.4, 2.8)	3.2 (2.6, 4.5)	*p* = 0.057
Sodium (mg)	320^b^	112 (65.3, 215.0)	200 (128.0, 312.0)	*p* = 0.065	218 (153.3, 345.3)	306 (236.0, 404.5)	*p* = 0.031
Calcium (mg)	525^b^	42.3 (18.0, 73.7)	135. (55.0, 240.1)	*p* = 0.002	288 (254.5, 324.0)	378 (306.5, 493.5)	*p* = 0.023
Vitamin D (µg)	8.5–10^c^^,^ d	0.1 (0.02, 3)	0.3 (0.1, 9)	*p* = 0.041	0.2 (0.1, 4)	0.99 (0.2, 5.2)	*p* = 0.009
Vitamin C (mg)	25^b^	6.8 (1.9, 14.5)	8.6 (5.1, 21.7)	*p* = 0.698	35.4 (30.3, 45.5)	43.0 (35.8, 65.5)	*p* = 0.229
Vitamin B_12_ (µg)	0.3–0.4^a^^,^ b	0.2 (0.1, 4)	0.34 (0.2, 9)	*p* = 0.060	0.2 (0.1, 4)	0.6 (0.2, 1.3)	*p* = 0.616
Folate (µg)	50^b^	15.8 (5.8, 23.2)	26.0 (11.9, 52.0)	*p* = 0.113	52.5 (40.7, 62.5)	67.0 (50.5, 97.5)	*p* = 0.083

Abbreviations: BLW, baby‐led weaning; TW, traditional weaning; RNI, recommended nutrient intake.

^a^ Dependent on age.

^b^ COMA 1991.

^c^ Safe intake.

^d^ SACN 2016.

For micronutrients, iron intake was significantly higher in the TW group both with and without milk intake included. However, neither group met the RNI for iron. Calcium and vitamin D intake from solid foods and the combined diet was significantly higher in the TW group. The only other difference in nutrient intake was noted for sodium, where intake was slightly higher in the BLW group when milk and solids were combined. Intake for all other nutrients was higher in the TW group. Both groups met the RNI for vitamins C, B_12_ and folate.

### Age group 2: 40–52 weeks old

Differences in overall estimated energy intake, macronutrients and micronutrients were analysed using MANCOVA controlling for timing of solid foods. Table 4 shows the WHO EAR for energy intake and the median estimated intake for infants in the two weaning groups for both solid foods and the combined diet. No significant difference was found in estimated intake between groups for either energy from solid foods alone or for solid foods and milk combined. In both calculations, both groups had an estimated median intake under the WHO EAR, although approxinately one‐third of infants consumed over the EAR.

**Table 4:** Comparison of estimated energy intake between weaning groups from solid foods and milk at 40−52 weeks using multivariate analysis of covariance and transformed data

		WHO EAR	Median intake (25th, 75th percentile)	% < WHO EAR	% > WHO EAR	Comparison of energy intake between groups
Solid food	BLW (*n* = 12)	1904 kJ	1427.0 (703.0, 1865.0) kJ	9 (75.0%)	3 (25.0%)	*F*_1,31_ = 830, *p* = 0.369
		455 kcal				
			341.0 (168.0, 445.8) kcal			
	TW (*n* = 24)		1674.0 (1152.0, 2198.0) kJ	16 (66.7)	8 (33.3%)	
			400.0 (275.3, 525.3) kcal			
Solid food + milk	BLW (*n* = 12)	3473 kJ	3117.0 (2298.0, 3646.0) kJ	8 (66.7%)	4 (33.3%)	*F*_1,31_ = 0.268, *p* = 0.608
		830 kcal				
			745.0 (549.3, 871.5) kcal			
	TW (*n* = 24)		3105.0 (2701.0, 3328.0)	16 (66.7%)	8 (33.3%)	
			742.0 (645.5, 795.5) kcal			

Abbreviations: BLW, baby‐led weaning; EAR, estimated average requirement; TW, traditional weaning; WHO, World Health Organization.

Table 5 shows the estimated intake of macro‐ and micronutrients for the two weaning groups. For both estimated macro‐ and micronutrient intake, no significant differences were found between groups either for solid foods alone or solid foods plus milk. Neither group met the RNI for iron, zinc, calcium or vitamin D, but both groups met the recommended intake for protein, sodium, vitamin C, B_12_ and folate and the intake of sodium was not at unhealthy levels.

**Table 5:** Comparison of estimated nutrient intake between weaning groups from solid foods alone and solid foods and milk at 40–52 week using multivariate analysis of covariance and transformed data

		Solids only			Solids and milk		
		BLW (*n* = 12)	TW (*n* = 24)		BLW (*n* = 12)	TW (*n* = 24)	
	RNI	Median (25th, 75th percentile)		*p*	Median (25th, 75th percentile)		*p*
Carbohydrate (g)	No RNI < 2 years	37.6 (21.9, 62.0)	50.5 (33.4, 68.8)	*p* = 0.367	84.0 (59.2, 100.5)	87.0 (71.7, 99.0)	*p* = 0.202
Protein (g)	12.7–13.7^a^^,^ b	14.3 (6.8, 16.6)	16.5 (10.5, 20.8)	*p* = 0.351	21.3 (13.9, 23.4)	22.5 (17.6, 26.9)	*p* = 0.437
Fat (g)	No RNI < 5 years	12.2 (5.8, 18.1)	11.9 (9.6, 16.6)	*p* = 0.652	35.3 (28.2, 41.5)	31.9 (28.8, 38.8)	*p* = 0.667
Saturated fat (g)	No RNI < 5 years	4.55 (2.4, 6.75)	4.8 (2.9, 7.5)	*p* = 0.517	15.01 (12.9, 17.3)	13.6 (12.2, 17.5)	*p* = 0.771
Sugar (g)	No RNI < 2 years	11.8 (7.1, 26.7)	20.3 (15.7, 26.1)	*p* = 0.066	50.5 (44.5, 67.2)	57.5 (48.2, 66.0)	*p* = 0.390
Free sugars	No RNI < 2 years	1.0 (0.5, 2.4)	1.5 (0.5, 2.5)	*p* = 0.715	1.0 (0.5, 2.4)	1.5 (0.5, 2.5)	*p* = 0.715
Fibre (g)	No RNI < 2 years	5.2 (2.1, 7.12)	5.8 (3.8, 7.4)	*p* = 0.771	6.1 (2.1, 8.6)	6.0 (3.6, 7.6)	*p* = 0.968
Iron (mg)	7.8^b^	1.8 (0.8, 2.6)	1.8 (1.1, 3.1)	*p* = 0.599	2.15 (1.2, 3.2)	3.1 (1.4, 4.9)	*p* = 0.525
Zinc (mg)	5^b^	1.6 (0.6, 2.2)	1.5 (0.87, 2.3)	*p* = 0.882	3.2 (2.2, 3.9)	3.3 (2.5, 4.1)	*p* = 0.890
Sodium (mg)	320–350^a^^,^ b	305 (205.5, 343.5)	288 (152.7, 398.5)	*p* = 0.605	396 (287.5, 469.2)	365 (246.3, 483.2)	*p* = 0.522
Calcium (mg)	525^b^	152 (61.7, 219.5)	205 (112.3, 278.7)	*p* = 0.108	332 (247.8, 405.5)	416 (353.8, 487.0)	*p* = 0.153
Vitamin D (µg)	8.5–10^c^^,^ d	0.34 (0.21, 44)	0.47 (0.1, 1.7)	*p* = 0.631	0.4 (0.2, 1.7)	1.3 (0.2, 4.0)	*p* = 0.608
Vitamin C (mg))	25^b^	14.7 (3.1, 32.1)	16.4 (9.3, 29.6)	*p* = 0.939	36.6 (24.7, 69.0)	45.5 (31.1, 58.5)	*p* = 0.923
Vitamin B_12_ (µg)	0.4^b^	0.65 (0.33, 1.1)	0.7 (0.4, 1.1)	*p* = 0.883	0.6 (0.3, 1.3)	0.9 (0.4, 1.2)	*p* = 0.933
Folate (µg)	50^b^	53.5 (19.8, 77.6)	38.2 (21.7, 65.0)	*p* = 0.336	91.0 (47.2, 117.3)	74.5 (51.2, 102.3)	*p* = 0.441

Abbreviations: BLW, baby‐led weaning; RNI, recommended nutrient intake; TW, traditional weaning.

^a^ Dependent on age.

^b^ COMA 1991.

^c^ Safe intake.

^d^ SACN 2016.

## DISCUSSION

Using 3‐day weighed diet records, the present study examined differences in the estimated energy and nutrient intake of babies aged 6–12 months dependent on their weaning approach. Overall, the findings showed the TW group consumed significantly more energy, carbohydrates and protein, alongside key micronutrients such as iron, calcium and vitamin D at 26–39 weeks, although there were no significant differences in estimated intake for infants 40–52 weeks of age in the two weaning groups. This suggests that, although differences in estimated energy and nutrient intake might be present at the start of weaning, they disappear as infants become more competent and start eating a larger proportion of solid foods in their diet. Notably, differences were more frequent when considering solid food alone compared to the cumulation of milk and solids together. Taken together, these findings have important considerations for health professionals supporting parents through the transition to solid foods and demonstrate the suitability of BLW as a method of complementary feeding as long as milk intake is maintained.

When looking at energy from complementary foods alone, the 26–39‐week TW group was consuming more than twice the calories of the strict BLW group from complementary foods, although a high degree of variability was seen. Most infants that were weaned using a strict BLW approach were eating under the recommended WHO guideline for complementary foods at the start of the weaning process, whereas the majority of TW babies were eating more than recommended. When solid foods and milk were considered together, the difference between the two groups was smaller yet still significant, with TW infants consuming around 10% more calories than BLW infants.

Comparatively, no significant differences were found in estimated energy intake between weaning groups for infants aged 40–52 weeks, either for solid foods alone or milk and solid foods taken together. Median consumption for both groups was under the WHO recommendation of 830 calories from milk and solids for infants of 9–11 months with one‐third of babies in each group consuming over the EAR. Likewise, this converging of energy intakes between BLW and TW infants was also found in the 24‐h recall reported by Pearce and Langley‐Evans.^12^

However, it is important to note that all infants were within healthy weight ranges, such that no infants were underweight despite a lower than recommended intake, which suggests that infants have a sufficient intake to meet their energy needs.^21^ Given the difficulty in estimating breastmilk (see below), it could well be that breastfed infants are consuming more than estimations used, and BLW were more likely to be breastfed. Likewise, no infant was overweight even when consuming more than recommended. However, weight can take time to incrementally increase and the longer term weight gain trajectories of infants consuming over the EAR would be useful to track. Notably, the BLISS study found that infants at 12 months consumed close to the WHO recommendations for infants aged 9–11months.^22^

Overall, BLW infants may on average be starting their transition to solid foods a little too slowly by WHO standards and the TW group too fast, but estimated intakes converge when infants are 40–52 weeks old. However, given the process of introducing solid foods to infants should be gradual, with an emphasis on continued milk intake, particularly in the early months, the findings highlight how BLW may support a gradual transition to solid foods. A slower transition to solid foods reduces the risk of the overconsumption of energy and macronutrients such as protein, or too fast a reduction in milk, which still provides significant nutrients and, in the case of breastmilk, antibodies and other protective factors.^23^ The baby‐led nature of feeding, where infants have greater control over intake, is likely responsible for this, alongside potentially a slower pace of meal as a result of self‐feeding.^24^ This may support infants in eating according to energy need rather than parental perceptions of need. However, parents should be supported practically to ensure that their infants *are* gradually making that transition and are not being offered *too few* solid foods.

There is little existing literature available describing energy intake of infants weaned using BLW for comparison. Neither of two New Zealand studies looking at infant weight^8^, ^25^ reported significant differences in energy intake between weaning groups. However, intention‐to‐treat analyses were used in the BLISS trial, with not all participants adhering to their allocated method, which could have affected intake. Also, potentially, the intake amongst BLW infants in the BLISS study could be affected by the trial protocol with respect to offering higher fat foods every day.

In the most recent UK 24‐h recall study,^12^ the results echoed our data in that TW infants aged 6–8 months had a higher energy intake from solid foods than BLW infants of the same age. However, in that study, there were no significant differences in overall energy intake (from milk and solid foods) at 6–8 months. Additionally, infants in both weaning groups on average consumed much greater amounts of energy from both milk and solid foods than in our study, notably at rates above the WHO EAR for all weaning groups. Likewise, in the other UK 24‐recall study, no difference in energy intake was found between weaning groups at either 6–8 or 9–12 months, although infants consumed a lower amount of energy from solid foods for both age groups than in the multiple pass recall study, again greater than the WHO EAR for all groups. Notably, however, in both these studies and as indicated in our data here, no differences in energy intake were seen at 9–12 months between weaning groups.

Why might infants in these two 24‐h recall studies be consuming more calories from solid foods than in the present study? Potentially, differences may arise as a result of the methods used. Weighed food diaries are likely to more accurately capture intake than recalls, which have been shown to overestimate intakes in infants and toddlers compared with weighed records.^26^ Indeed, infants in our study were eating a comparably closer amount to infants in the BLISS RCT study which also used a weighed food diary.^27^ Directly measuring and weighing foods may also make a difference particularly for infants at this age because the amounts consumed are relatively small to begin with.^13^, ^14^

For macronutrient intake, several differences occurred between the two younger weaning groups although these disappeared towards the end of the weaning process, following the same pattern as in the recent 24‐h recall study.^12^ These initial differences can partly be explained by disparities in overall intake between the two groups. If the overall food intake is higher, levels of macronutrients in that food will also be higher; thus, the TW group took in over twice the median energy of the BLW group when solid foods alone were examined, with the expectation that they would also have a similarly increased intake of macronutrients.

For both milk and solids together, at 26–39 weeks, the TW group had a median estimated carbohydrate intake 29% higher than the BLW group and a protein intake that was 36% higher. However, similar to the BLISS study,^8^, ^22^ the BLW group had a median estimated fat intake almost identical to the TW group, meaning their diet was more fat dense given the lower volume of solid foods consumed. Higher fat intake in BLW infants could be attributed to a higher milk intake because breast and formula milk have relatively high fat contents compared to many weaning foods. This was the case in the recent UK 24‐h recall study^12^ with a higher milk intake raising fat content consumption in younger BLW infants.

However, it could also be a result of the type of food consumed, particularly in the older age group, where estimated fat intake matched the TW group for solid foods alone. Previous research has shown that TW infants eat more commercially prepared composite infant meals, whereas BLW infants are more likely to eat family foods.^11^, ^28^, ^29^ Commercial infant meals tend to be higher in sugars and starchy carbohydrates but lower in fat compared to average family meals.^30^ It may also be a consequence of health professional concerns that infants may not eat sufficient energy if parents are using BLW,^2^, ^3^ leading parents to potentially offer higher fat foods. Indeed, in the BLISS study, the protocol was designed to meet these concerns, encouraging parents to offer healthy fat‐rich foods every day.

However, given that only overall estimated fat intake was significantly different and not saturated fat, as well as the small amounts involved and the importance of fats in growth and development,^31^, ^32^ this should not be viewed as a negative finding, especially because the difference disappeared in the older group. Estimated intake of fat was also weighted towards unsaturated fats found in avocado and fish rather than saturated fats found in processed foods. Indeed, the higher estimated protein intake of the TW infants may be more problematic as a result of its association with rapid growth and increased fat deposition.^33^ In this instance, although the estimated protein intakes of both groups of older infants were in excess of recommendations, when considered as a percentage of energy intake, neither groups consumed more than 13% of their energy from protein, which is not a risk factor for later obesity according to prior research.^34^ This was also seen in the younger age group, where, although estimated intake was above UK government recommendation, the mean estimated intake of protein as a percentage of energy was not concerning.

Turning to micronutrients, estimated iron intake was significantly lower in the BLW group at 26–39 weeks. This could be a result of the increased use of iron‐fortified formula or cereals in the TW group, although both groups consumed less than the RNI. However, iron *intake* does not necessarily equate to differences in iron *absorption*. Infant formula is fortified with iron to levels above those seen naturally in breast milk, but it is absorbed at a lower rate,^35^ as is the non‐haem iron in infant cereal.^36^, ^37^ Meanwhile, meat, fish or poultry in a mixed meal increases the absorption of any non‐haem iron present,^37^, ^38^ whereas phytates (found in whole grains) and calcium inhibit absorption.^36^ The lower dietary calcium in the BLW group may thus have had a positive impact on iron absorption.

Research examining iron intake by weaning approach is mixed. Although one New Zealand study found a lower iron intake in BLW infants,^8^ the BLISS study did not.^22^ However, both studies found that infants were consuming below recommended levels. Conversely, in the Turkish randomised controlled trial, no difference was found at 12 months with respect to iron intake from complementary foods or hematologic markers.^9^ However, parents who were randomised to the BLW group received advice on high‐iron and energy‐dense foods and recipes, highlighting the importance of messaging for parents following the approach. Finally, both UK 24‐h recall studies found low levels of iron consumption, with BLW infants consuming lower amounts than TW infants. This was attributed to a lower intake of iron fortified cereal in BLW infants, although it was also noted that lower levels of iron in breastmilk may skew this issue.^10^, ^12^ As noted above, amounts may be lower but absorption occurs at a greater level. Further research is needed into iron status of infants rather than considering intake alone.

There are limitations to the present study. The sample used was self‐selecting both in terms of participation and weaning approach and, in all likelihood, comprised a highly motivated and well‐educated cohort with over 80% of the respondents holding at least a University degree. Further research is needed within a more diverse sample. We also excluded those infants with developmental or health issues, meaning that our findings are not applicable to all and caution should be applied in following a BLW method for those infants who are not thriving or able to self‐feed.^39^

Other limitations include possible participant error or inaccuracy with respect to measuring or recording foods, particularly left overs when foods are self‐fed. This is a common limitation of any diet diary or food recall study. Although detailed instructions were given, some parents simply stated that their infant had eaten a family meal such as “Spaghetti Bolognese” without giving the exact recipe, such that estimates had to be made using the Nutritics database. We also did not collect or record vitamin supplements because it is difficult to estimate absorption/efficacy rates. There are also limitations to the Nutritics software, which has some missing data on micronutrients for manufactured foods. Together, this means that micronutrient amongst infants may have been underestimated.

Another important limitation is the attempt to accurately measure breastmilk intake. We initially asked mothers to record how many feeds they gave and to estimate duration. However, the returned data was very patchy, especially for night feeds. It also did not take into account variation in energy density of milk between mothers and speed of milk delivery (i.e., milk ejection speed, strength of suck), which could alter how much milk two infants receive within the same period of time.^40^ As per the BLISS study, we used average intake calculated in previous studies based on test weighing and isotopes,^15^, ^41^ although it is likely that these intakes were based on infants who were spoon‐fed. Breastmilk intake may be different for infants who are self‐feeding and further research is needed. Furthermore, as noted above, given that no infant in the study was underweight, it was likely that breastfed infants consuming a lower level of energy from solid foods were meeting their energy needs from increased breastmilk consumption.

Overall, this is the first study of its kind in the UK to investigate a weighed food record and the detailed estimated nutrient intake of babies weaned using a strict form of BLW. It illustrates that few differences occur in overall nutrient intakes between BLW and TW infants, especially in the later stages of weaning. It also highlights that all parents may need further support, particularly around offering iron rich foods, regardless of weaning approach.

## CONFLICT OF INTERESTS

The authors declare that there are no conflicts of interest.

## ETHICS STATEMENT

Approval for this study was granted by Swansea University College of Human and Health Research Ethics Committee. All mothers gave informed consent prior to inclusion in the study.

## TRANSPARENCY DECLARATION

The lead author affirms that this manuscript is an honest, accurate, and transparent account of the study being reported. The reporting of this work is compliant with STROBE. The lead author affirms that no important aspects of the study have been omitted and that any discrepancies from the study as planned have been explained.

## AUTHOR CONTRIBUTIONS

Hannah Rowan was responsible for the study design, data collection, data analysis, draft report writing and critical revisions. Michelle Lee was responsible for critical revisions. Amy Brown was responsible for study design, data analysis support and critical revisions.

## Supporting information

Supporting information.

## Data Availability

Data are available upon reasonable request from the authors.
