# Supplementary material for: Estimated energy and nutrient intake for infants following baby‐led and traditional weaning approaches
Source: J Hum Nutr Diet. 2022 Jan 27;35(2):325–36. doi: 10.1111/jhn.12981 (PMC9511768; doi:10.1111/jhn.12981)
Supplement: Supplementary file 1 — Supporting information. [file JHN-35-325-s001.docx]

**Supplementary Appendix**

**Figure 1: Example of a weighed three day diet diary**

| **Step 1** | **Step 2** | | | **Step 3** | | **Step 4** | | | | **Step 5** | | | **Step 6** | |
| --- | --- | --- | --- | --- | --- | --- | --- | --- | --- | --- | --- | --- | --- | --- |
| Time of day | Name of food or drink | Brand of food or drink | Cooking method | Weight of plate/mug | Weight of food/drink and plate/mug | Consistency of food/drink | | | | Food was placed into the child’s mouth by: | | | Weight of leftovers + plate or mug | Estimate how much is left on plate/mug |
|  |  |  |  |  |  | Pureed | Mashed | Diced | Whole | Adult | Child | Mixed |  |  |
| 6.30am | Breastfeed for 20 minutes |  |  |  | . |  |  |  |  |  |  |  |  |  |
| 8.30am | Breastfeed for 15 mins |  |  |  |  |  |  |  |  |  |  |  |  |  |
|  | White toast & | Hovis |  | 50g | 75g |  |  |  | X |  | X |  |  | Half |
|  | Butter | Tesco |  |  |  |  |  |  |  |  |  |  |  |  |
|  | Banana – half |  |  |  | 175g |  |  |  | X |  | X |  | 70g | None |
| 10.30am | Breastfeed for 10 minutes |  |  |  |  |  |  |  |  |  |  |  |  |  |
| 12pm | Ella’s Kitchen carrot crunchy snacks –bag (at café) | Ella’s |  |  | 15g |  |  |  | X |  |  | X |  | Half |
|  | Strawberries (at café) |  |  |  | 3 large |  |  |  | X |  | X |  |  | None |
|  | Water (at café) |  |  |  | 4 sips |  |  |  |  |  |  |  |  |  |
| 3pm | Fromage frais small carton | Tesco |  |  | 55g |  |  |  |  |  |  | X |  | None |
| 4pm | Breastfeed 20 mins |  |  |  |  |  |  |  |  |  |  |  |  |  |
| 6pm | Fishfinger, cod | Tesco | Grilled | 50g | 110g |  |  |  | X |  | X |  |  | None |
|  | Potato, 1 small |  | Boiled |  | 185g |  | X |  |  |  | X |  |  | Most |
|  | Frozen peas | Aldi | Boiled |  | 215g |  |  |  | X |  | X |  | 125g | Half |
|  | Water |  |  | 100g | 200g |  |  |  |  | X |  |  | 180g | Most |
| 7pm | Breastfeed 15 mins |  |  |  |  |  |  |  |  |  |  |  |  |  |
